# Topo2*α* protein expression predicts response to anthracycline combination neo-adjuvant chemotherapy in locally advanced primary breast cancer

**DOI:** 10.1038/sj.bjc.6605960

**Published:** 2010-11-09

**Authors:** A Mukherjee, M Shehata, P Moseley, E Rakha, I Ellis, S Chan

**Affiliations:** 1 Department of Oncology, Nottingham University Hospitals, City Hospital Campus, Hucknall Road, Nottingham, NG5 1PB, UK; 2 Department of Pathology, Nottingham University Hospitals, City Hospital Campus, Hucknall Road, Nottingham, NG5 1PB, UK

**Keywords:** biomarkers, neo-adjuvant therapy, breast cancer

## Abstract

**Background:**

This study aimed to identify predictors of response to anthracycline-based chemotherapy (5-fluoro-uracil, epirubicin, cyclophosphamide (FEC)) in locally advanced primary breast cancer (LAPC).

**Methods:**

A total of 91 LAPC patients were treated with six cycles of FEC before surgery. Protein expression of nine biomarkers (topoisomerase2*α* (Topo2*α*), ER, PR, HER2, Ki67, p53, EGFR, CK5/6 and CK14) was assessed in pre-chemotherapy core biopsies using immunohistochemistry (IHC) and results correlated with clinical and pathological response.

**Results:**

Clinical (cCR) and pathological (pCR) complete response were seen in 34.1% (*n*=31) and 20% (*n*=18), respectively. Pathological complete response was concordant with cCR in 14/31 cases; in four cases of cPR with palpable residual breast tumours, histology showed fibrous tissue only (pCR). On univariate analysis, pre-chemotherapy high expression of Topo2*α* protein (*P*=0.031), and negativity for ER and EGFR (*P*=0.001 and *P*=0.005, respectively) correlated with pCR. Positivity for p53 also showed significance (*P*=0.015), whereas basal phenotype, HER2, and all the clinicopathological variables of LAPC included in this study did not show significant correlation with response. On multivariate analysis, Topo2*α* expression had the strongest correlation with pCR (*P*=0.021) followed by EGFR (*P*=0.044).

**Conclusion:**

The study suggests that pre-chemotherapy Topo2*α* protein expression measured by IHC strongly correlates with pathological CR to neo-adjuvant anthracyclines in this group of LAPC studied.

Neo-adjuvant chemotherapy is an established treatment modality for locally advanced primary breast cancer (LAPC) (Mieog *et al*, 2007). It aims to reduce the size of the primary tumour, to enable complete surgical removal of the primary tumour and axillary lymph-node metastases, and in smaller/earlier tumours it may allow breast conservation and other oncoplastic options without compromising tumour control. In addition, serial biopsies taken during neo-adjuvant chemotherapy offers the chance of individualization of therapy by offering the scope of evaluating early molecular changes of various biological or pathological markers predictive of response or resistance.

The optimal neo-adjuvant combination for the individual patient is a worthy research question. Anthracycline-based regimens are commonly used because it is one of the most effective in this setting. The reported clinical (cCR) and pathological (pCR) complete response rates for anthracycline alone and anthracycline combination regimes are in the range of 30–40% and 13–26%, respectively (Mieog *et al*, 2007). Furthermore, there are about 5% of breast cancers, which are resistant (have clinically progressive disease) to neo-adjuvant anthracycline chemotherapy. In these cases, unnecessary side effects such as cardio-toxicity may be avoided if biomarkers of response are identified.

Integral to the action of anthracyclines is the inhibition of topoisomerase2*α* (Topo2*α*). This subclass of topoisomerase binds DNA and forms transient double-stranded breaks in replicating DNA, allowing passage of a second DNA double-helix strand. Inhibition of Topo2*α* leads to double-strand DNA breaks and cell death (reviewed by Di Leo *et al*, 2008). Previous studies have demonstrated that Topo2*α* is a predictor of response to anthracycline-based therapy (MacGrogan *et al*, 2003; Durbecq *et al*, 2004). However, other studies have indicated that other markers such as HER2 (Sánchez-Muñoz *et al*, 2008) or basal phenotype (BP) (Rakha *et al*, 2008) may provide predictor information. Proliferation as assessed by Ki67 has also been shown to correlate with outcome (Bozzetti *et al*, 2006). Therefore, in this study we assessed the predictive value of multiple molecular biomarkers in a series of 91 patients presenting with LAPC who had been offered anthracycline-based therapy.

## Materials and methods

### Patient treatment profile

A total of 91 patients presenting with LAPC between December 1996 and December 2009 at our institution and treated with neo-adjuvant anthracycline-based chemotherapy were included in this study. A core biopsy was performed before the chemotherapy treatment to allow a pathological diagnosis and evaluation of biological parameters. Patients were treated with the following regimen.

Six cycles of an anthracycline-based therapy (FEC: 5-fluorouracil (5-FU) 500 mg m^−2^, epirubicin 75–100 mg m^−2^, cyclophosphamide 500 mg m^−2^, on day 1 of a 21-day cycle). Patients were scheduled to undergo surgery 4 weeks after the sixth cycle.

### Assessment of response

Assessment of the tumour response was undertaken by clinical measurements both before chemotherapy treatment and after each cycle of chemotherapy. The clinical baseline and preoperative measurements were obtained with a calliper by the same clinician or by radiological assessment. Clinical response was recorded according to RECIST criteria (Park *et al*, 2003a).

The pathological response was evaluated by histological examination of tumour removed following chemotherapy. The surgical procedures that were performed included the following: Simple mastectomy (13.7%), Patey mastectomy (82.9%) and wide local excision (3.4%). Histology including grade was reviewed and agreed upon by two experienced breast pathologists utilising the Chevallier classification: the absence of invasive tumour cells or persistence of only *in situ* disease, and negative axillary lymph nodes define a pathological CR (Chevallier *et al*, 1993). On an average, 16 breast blocks and all submitted lymph nodes (in entirety) were examined for each case before a diagnosis of pCR was reached.

### Immunohistochemistry techniques for biomarkers

Immunohistochemistry was done on core biopsies (pre-treatment) with the use of a standard avidin–biotin peroxidase technique (Madjd *et al*, 2003). Heat-induced epitope retrieval consisting of 20-min microwave treatment (10 min at high power and 10 min at low power) in pH 6.0 citrate buffer was necessary for all markers, except HER2. For Ki67 staining, sections were treated with 10% trypsin before heat-induced antigen retrieval. Sources and dilution of primary antibodies are summarised in Table 1.

### Evaluation of staining

Two observers, blinded to the clinicopathological data, evaluated the staining for consensus. Cut-off values for scoring are summarised in Table 1. The cut-off values were chosen as per standards peer-reviewed and well accepted in literature (Rakha *et al*, 2006, 2007). For Topo2*α*, the median was chosen as the cut-off for high and low staining as the data distribution is not homogeneous (Supplementary Figure 1).

### Statistical analysis

Statistical analysis was performed using SPSS17 statistical software (SPSS Inc., Chicago, IL, USA). Pearson's *χ*^2^-tests were used to determine the significance of associations between categorical variables. Overall survival (OS) calculations included all patients who died during follow-up. Survival rates were calculated using the Kaplan–Meier method; differences between groups were tested using the log-rank test. Events for relapse-free and OS were defined as follows: time of disease relapse, either local or distant (for relapse-free survival), and time of death, (for OS), respectively. The Cox proportional-hazards model was used for multivariate analysis in order to determine the relative risk and independent significance of individual factors. In all cases two-sided *P*-values of <0.05 were considered as statistically significant.

## Results

### Clinicopathological characteristics

The mean age of the patients was 51 years (range 25–76 years). Histologically, the tumours were classified as follows: Ductal NST 83 (91.2%); lobular (8.8%). The tumours were T's taged as follows: T2 14 (15.6%); T3 72 (79.2%); T4 5 (5.2%). Four of these were inflammatory breast cancers. The grades of the tumours were as follows: G1 1 (1%); G2 40 (44%); G3: 50 (55%). The N-stage for such cases treated by neo-adjuvant therapy may not be very accurate as it is based on clinical examination and not all palpable lymph nodes may be involved by tumour. Hence this was not included in the paper. By definition, the M status of locally advanced breast cancer patients is M_0_ and this was confirmed in the current series.

The characteristics of the tumours analysed by IHC are summarised in Table 2.

### Response to chemotherapy regimens

The overall response to anthracycline combination was as follows: cCR 31 (34.1%); cPR 42 (46.2%) (cORR 80.3%); cSD 15 (16.4%); cPD 3 (3.3%). Pathological complete response was observed in 18 patients (20%). Clinical CR conformed to pCR in 14 cases; in 4 cases of clinical partial response, histology showed fibrous tissue only (pCR).

### Biomarker expression

#### Immunohistochemistry results.

The relationship of immunohistochemical markers and pCR are summarised in Table 2 (and Supplementary Table2). On univariate analysis, the following biomarkers correlated with pCR to anthracyclines: Pre-chemotherapy high Topo2*α* expression (*P*=0.031), ER– (*P*=0.001), EGFR− (*P*=0.005) and p53+ (*P*=0.015). Patient age (*P*=0.65), tumour size (*P*=0.7), histological subtype (*P*=0.58), tumour grade (*P*=0.112), HER2 status (*P*=0.354), progesterone receptor status (*P*=0.116) and Ki67 (*P*=0.71) did not predict response to anthracycline-based therapy. There was no correlation between Topo2*α* protein expression and HER2 protein expression in the examined series (*P*=0.692). Neither was there any correlation between Topo2*α* and Ki67 (*P*=0.9) in this study.

For this study, the BP was defined as immuno-phenotypic evidence of basal cytokeratins CK5/6 and/or CK14 expression (>10%) (Rakha *et al*, 2006). The BP was highly expressed in 48 patients (52.7%). The BP status was not significant for response to neo-adjuvant chemotherapy (*P*=0.26). When defined as BP and EGFR+, the incidence of the BP is 23.9% this was also not significant for response to neo-adjuvant chemotherapy (*P*=0.5) (Nielsen *et al*, 2004). In all, 44% of the BP+ cases are p53 positive. This is however not statistically significant. The incidence of triple-negative cancers in the series is 24.1%.

Multivariate analysis was conducted including ER status, EGFR status, p53 and Topo2*α* protein status as variables in the regression equation. Topo2*α* protein expression correlated with pCR (*P*=0.021; estimated HR: 6.587; 95% confidence intervals (CI): 1.327–32.707; of the other variables, EGFR– status was also significant (*P*=0.044; estimated HR; 4.765; 95% CI: 1.045–21.729). Both ER (*P*=0.64) and p53 (*P*=0.422) failed to achieve significance.

### Survival analysis

**Figure 1:**
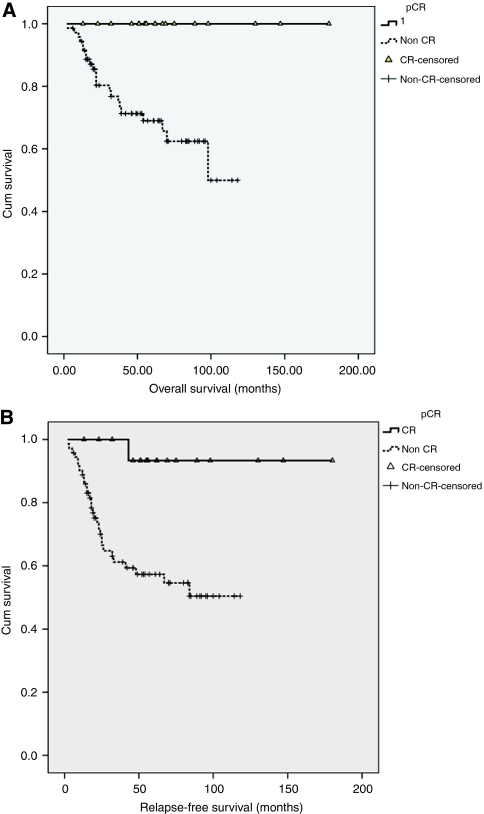
Overall survival (**A**) and relapse-free survival (**B**) analysis for patients with pCR and non-pCR to anthracyclines.

Pathological complete response for the series correlated with better relapse-free (*P*=0.005) and OS (*P*=0.006) (Figure 1A and B). A total of 31 patients included in this study had relapsed after their neo-adjuvant chemotherapy; the median follow-up was 62 months. A total of 21 patients have died from breast cancer during the period. The estimated mean RFS for those who achieved pCR was 140 months. For those who had progressive disease, the estimated median OS was 7 months.

**Figure 2:**
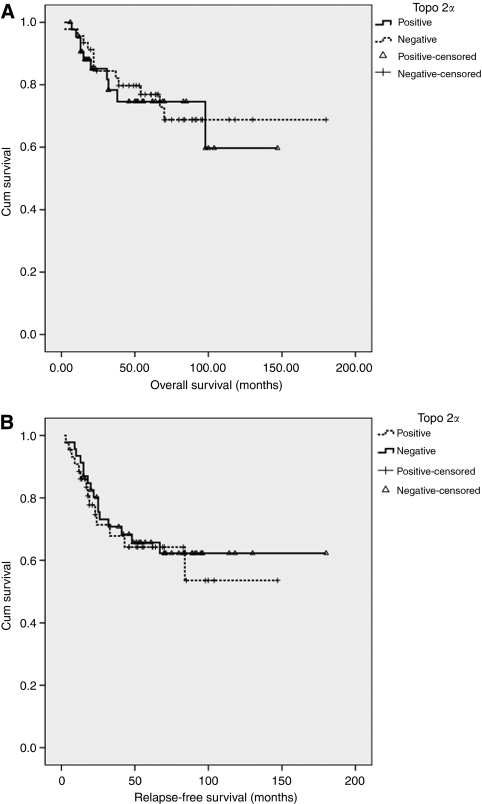
Overall survival (**A**) and relapse-free survival (**B**) analysis for patients with low and high Topo2*α* protein levels.

**Figure 3:**
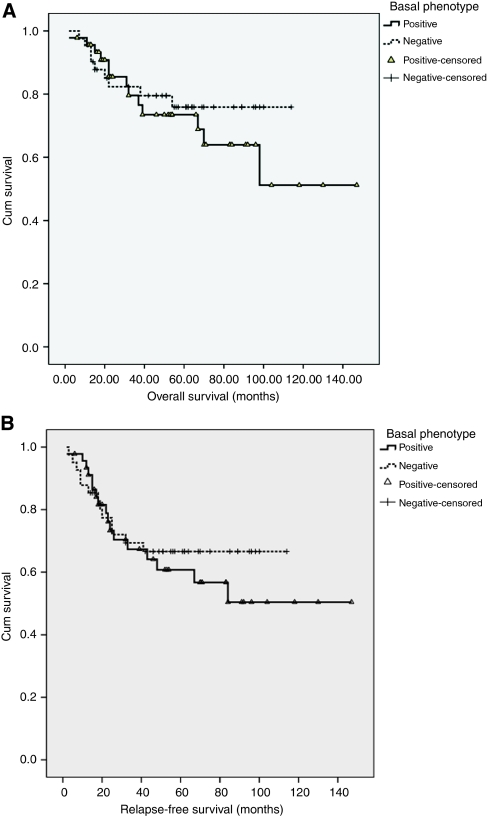
Overall survival (**A**) and relapse-free survival (**B**) analysis for patients either positive or negative for basal phenotype (BP).

Of the relapses and deaths, 19 (61%) and 10 (47%) were Topo2*α*-high, respectively. However, Topo2*α* status did not predict either relapse-free (*P*=0.67) or OS (*P*=0.76) (Figure 2A and B). Similarly, although patients with BP tumours tend to have a worse OS and RFS, this was not statistically significant (*P*=0.5 and *P*=0.54, respectively) (Figure 3A and B).

## Discussion

Neo-adjuvant chemotherapy for patients with LAPC has been shown to increase surgical resectability and breast conservation rates (Mieog *et al*, 2007). A unique advantage of primary chemotherapy is the possibility to take serial measurements and biopsies of the primary tumour, thus allowing an *in vivo* assessment of factors predictive of response or resistance to treatment.

The use of anthracycline regimen in the adjuvant setting has reduced mortality due to breast cancer (Cardoso and Piccart, 2003), but significant cardiac toxicity leading to congestive cardiac failure has been reported in 3.7% of patients treated with doxorubicin (Chan *et al*, 1999). It is clear that the use of the drug should be avoided in resistant tumours. Hence, the search for biomarkers predictive of resistance to neo-adjuvant anthracycline containing regimes is important.

Pathological complete response is the most reliable end-point of response to neo-adjuvant treatment and hence was chosen in this study. Pathological complete response for the series correlated with better OS. Factors associated with pCR on univariate analysis include Topo2*α*, EGFR, ER and p53.

The evidence in literature for and against Topo2*α* as a marker for response to anthracyclines is variable. The main role of Topo2*α* has been explored as a prognostic and predictive biomarker for anthracyclines in the adjuvant setting and the results thus have been far contradictory. This study is unique in that it deals with a pure cohort of patients of locally advanced breast cancer who did not have any treatment (surgery, radiotherapy, chemotherapy or hormonal therapy) before anthracycline chemotherapy.

This study evaluated Topo2*α* protein expression as adjudged by IHC. Previous studies looking at anthracyclines and Topo2*α* have utilised different end-points *viz*. gene expression or protein analysis. Topo2*α* is regulated at multiple levels, *viz.* at the gene level (amplification and deletion), post-translational mechanisms such as mRNA stabilisation, subcellular protein distribution and isoform expression. Immunohistochemistry evaluation of Topo2*α* though criticised as being semi-quantitative is a holistic end-point. Protein expression summates the net-effects of gene translational and proliferation controls, and hence was chosen for this study. Only nuclear staining was considered positive, taking into account the active subcellular location. Tumour-proliferation status is known to have a key role in regulating Topo2*α* protein levels, independently of *Topo2α* gene status (Di Leo *et al*, 2008). In our series, however, no correlation was observed between Topo2*α* and Ki-67 protein levels and differential chemosensitivity does not appear to be simply a function of the proliferation status of the tumour. It is important to stress here that the LAPC cohort represents a highly selected population with most tumours being either grade 2/3 (98%) and having a high proliferation index (79% with Ki67 >10%).

Our results showed that Topo2*α* protein as measured by IHC in pre-chemotherapy tumours strongly correlates with pCR. This concords with the results reported previously (MacGrogan *et al*, 2003; Martin-Richard *et al*, 2004) that show that the response to neo-adjuvant chemotherapy was correlated with the IHC expression of Topo2*α*. Recent reports in the adjuvant setting also confirm that Topo2*α* protein rather than gene expression correlates with response to anthracycline-based therapy (Schindlbeck *et al*, 2010). Park *et al* (2003b) reported a good response to doxorubicin in breast cancers with co-amplification of HER2 and Topo2*α* (as measured by chromogenic *in situ* hybridisation) and in HER2-amplified tumours with or without Topo2*α* amplification. Co-amplification of HER2 and Topo2*α* also correlated with response to anthracycline-based therapy in a series of high-risk primary breast cancers (Konecny *et al*, 2010). In our studies, there was no correlation between HER2 and Topo2*α* expression as measured by IHC, and HER2/Topo2*α* co-expression on a protein level did not correlate for response to anthracyclines. Recent reports from the MA.5 trial show that Topo2*α* protein overexpression is not closely correlated to HER2 positivity but is a significant predictor of differential response to anthracycline combination chemotherapy (O’Malley *et al*, 2006). This study did not preselect for HER2-positive patients treated with anthracyclines. We concur with the view that the ‘jury is still out’ over this issue with both proponents and opponents for such selection (Esteva and Hortobagyi, 2009).

The predictive value of Topo2*α* protein levels in the neo-adjuvant setting in locally advanced breast cancer parallels other retrospective studies in early or advanced breast cancer. In the adjuvant setting, a dose-intense anthracycline-based chemotherapy showed superiority over a less intensive regimen only in the cohort of patients carrying Topo2*α* protein overexpression (Di Leo *et al*, 2001). Results from a retrospective phase III clinical trial conducted by the National Cancer Institute – Canada Group also confirm that Topo2*α* protein overexpression might be associated with increased benefit from doxorubicin (hazard ratio 1.09, 95% CI: 1.03–1.15, *P*=0.002) (Durbecq *et al*, 2004).

The prospective TOP trial that was specifically designed to identify markers of response/resistance to preoperative epirubicin in oestrogen receptor-negative breast cancer patients, showed that Topo2*α* amplification (as identified by FISH) was exclusively observed in HER2-amplified cases and was highly predictive of pCR (Desmedt *et al*, 2009). In contrast to this study, protein levels were not predictive of pCR. In another small series of 23 patients with endocrine unresponsive and HER2-overexpressing tumours, Topo2*α* amplification correlated with a significantly high probability of achieving pCR after neo-adjuvant, anthracycline-based chemotherapy (Orlando *et al*, 2008).

In spite of being correlated with pCR, Topo2*α* protein levels did not show any particular trend in the cases that had progressive disease. Notwithstanding the small number of cases with progressive disease, it appears that although the Topo2*α* level predicts for response, it does not automatically predict for the most resistant. Tumour resistance depends on complex interaction of multiple factors. Similarly, the lack of significant correlation between overall or progression-free survival withTopo2*α* may also be attributed to the interplay of multiple factors.

Apart from Topo2*α*, the EGFR– status was predictive of pCR in univariate and multivariate analysis in this study. In other studies, EGFR-negative tumours had a better OS following neo-adjuvant anthracyclines but did not predict for pCR (Buchholz *et al*, 2005 and Schippinger *et al*, 2007). The association of the EGFR− status with pCR in this study may be explained by the downstream signaling pathways activated by EGFR signaling resulting in resistance to chemotherapy-induced apoptosis.

In our study, the ER negative status is an indicator of response to anthracyclines on univariate analysis. In a larger series of 485 patients with LAPC (cT2-T4, N0-2, M0) ER/PR− tumours were 12 times more likely to achieve a pCR to anthracyclines (*P*<0.0001) (Colleoni *et al*, 2008). Thus the ER negative status may be an indication of response to pre-operative chemotherapy in LAPC.

The p53 is a known tumour suppressor and the nuclear protein has an essential role in the regulation of cell cycle. Breast cancer patients with p53 mutations or protein accumulation have been shown to have worse survival (Miller *et al*, 2005). Pathological complete response was associated with overexpressed p53 in our series, as reported in other studies (Colleoni *et al*, 1999; Faneyte *et al*, 2003; Mieog *et al*, 2006). Recent findings (Bertheau *et al*, 2007) indicate that non-inflammatory breast tumours containing mutant TP53—in particular, basal-cell-like tumours—are very sensitive to dose-dense epirubicin and cyclophosphamide chemotherapy. The correlation between positive p53 and response has been explained by the dose-dense regime used in the study. Other authors (Faneyte *et al*, 2003) have tried to explain the association of p53+state with pCR with the following hypothesis: possibly, the DNA damage that is initially tolerated by cells with mutated p53 (positive on IHC) decreases viability during subsequent tumour proliferation. In contrast, with intact p53 (negative on IHC), DNA repair mechanisms rescue cancer-cell viability. It must be remembered that the correlation between p53 protein accumulation and the presence of mutations is not absolute and hence, IHC results must be interpreted with caution.

A recent progress into the molecular classification of breast cancer includes the BP, originally identified from its gene-expression profile. Our study used the stated definition of the BP and found a high propensity of the cytokeratins in our LAPC tumours. The high incidence probably reflects the highly selected group of locally advanced breast cancers, which might have an incidence of BP different from the whole breast cancer population. It is sometimes perceived that BP tumours and triple-negative tumours are synonymous and BP can be defined using a triple-negative definition without the need for the expression of basal markers. However, contrary to popular belief, it has been shown that triple-negative tumours are a heterogeneous population that may either be basal cytokeratin-positive or basal cytokeratin-negative (Rakha *et al*, 2009). Hence, the basal cytokeratin-based definition that has been widely used in the literature (Rakha *et al*, 2006, 2008) and in this study is probably more relevant in the authors’ opinion.

As for response to anthracycline-based chemotherapy, the BP status did not predict response/resistance to anthracyclines in our series. The response of basal-like tumours to chemotherapy is not well-defined (Rouzier *et al*, 2005; Rodríguez-Pinilla *et al*, 2006; Banerjee *et al*, 2006; Tan *et al*, 2008). In one study (Carey *et al*, 2007), the basal-like and HER2 subtypes, defined by hormone receptor IHC only, had the highest rates of pCR to pre-operative anthracycline-based chemotherapy, whereas the luminal tumours responded poorly. Paradoxically, despite the significantly higher prevalence of pCR in the basal-like group, patients with these tumours still displayed the worst outcome. These discrepancies might reflect differences in defining the BP, chemotherapy regimens or patient populations studied, protein expression *vs* gene profiling, as well as the molecular heterogeneity of basal breast tumours. As for overall and progression-free survival, the BP+-cohort tends to fare worse. The BP+ group inherently defines cancers of a bad prognostic group, documented well in the literature.

This study concludes that high Topo2*α* is the biomarker that has the strongest correlation with pCR to neo-adjuvant anthracycline combination. The strength of this study is the fact that these patients had no previous cancer treatment, therefore the pCR rate can be directly attributed to the FEC chemotherapy. Furthermore, a battery of biomarkers were examined concurrently with Topo2*α* and preselection based on HER2 status, was avoided. Given the controversies surrounding the best and most accurate way to assess the *Topo2α*-gene expression, the authors suggest that the protein level, as determined by IHC, is an easily available as well as summative end-point that should not be disregarded in future studies. Further proteomic fine-tuning of quantitative analysis for Topo2*α* protein levels may help clinicians tailor their selection of chemotherapeutic agent for neo-adjuvant treatment. The alkylating agent administered with the anthracyclines may also be a determinant of response and hence these associations should be explored further. Other markers associated with pCR status are also emerging for example, stromal gene signatures (Farmer *et al*, 2009) and the presence of tumour infiltrating lymphocytes (Denkert *et al*, 2010). A combined genomic and proteomic analysis on tissue and serum samples obtained sequentially during neo-adjuvant chemotherapy may help define the molecular response pathway, which can be utilised to select the optimal treatment for the individual patient.

## Figures and Tables

**Table 1:** Source, dilution and cut-off values for positivity for different biomarkers

**Marker**	**Antibody (Source)**	**Optimal dilution**	**Positive-control tissue**	**Cut-off values for positivity**
ck5/6	D5/16 B4 (Dako UK Ltd, Ely, Cambridgeshire, UK)	1 : 100	Tonsil	10%
ck14	LL002 (Vector Labs, Peterborough, UK)	1 : 100	Skin	10%
ER	1D5 (Dako UK Ltd)	1 : 100	Breast	1%
PGR	PGR 636 (Dako UK Ltd)	Neat	Breast	1%
HER2	DakoA0485 (Dako UK Ltd)	1 : 500	Breast	30% membranous (corresponds to IHC 3+), or if IHC 2+, confirmed by FISH+
p53	DO7 (Dako UK Ltd)	1 : 100	Breast	10% nuclear
EGFR	EGFR.113 (Dako UK Ltd)	1 : 50	Skin	10% membranous
Topo2*α*	KI-S1 (Dako UK Ltd)	1 : 100	Tonsil	45% nuclear (high)
KI67	MIB1 (Dako UK Ltd)	1 : 100	Tonsil	10% nuclear (high)

Positive-control tissue is also indicated. Positive HER2 was defined as presence of membrane expression of the protein in >30% of tumour cells (corresponds to IHC 3+) or if underexpressed (2+), confirmed by FISH (fluorescent *in situ* hybridisation). For p53, Topo2α and Ki67, 100 nuclei were scored for positivity in four random fields ( × 40 magnification) and the total percent-score obtained. Median values of Topo2α were chosen as cut-offs for high or low expression. Other standard cut-offs as per Rakha *et al* (2006, 2007).

**Table 2:** Immunohistochemistry analysis and correlations with pCR for patients treated with anthracyclines

**Parameter**	**Variables**	**%**	**%pCR**	**%non-pCR**	***P*-values**	**Odds ratio**
ER status	Positive	51.6	6.4	93.6	0.001	0.132 (0.35–0.496)
	Negative	48.4	34.1	65.9		
PR status	Positive	36.3	12.1	87.9	0.116	2.636 (0.765–9.080)
	Negative	49.4	26.7	73.3		
	Missing	14.3	NA	NA		
HER2 Status	Positive	29.7	14.8	85.2	0.354	0.562 (0.164–1.923)
	Negative	60.4	23.6	76.4		
	Missing	9.9	NA	NA		
Topo2*α* status	High	51.6	28.9	71.1	0.031	3.331 (1.076–10.315)
	Low	48.4	13.9	86.1		
EGFR status	High	63.5	12.5	87.5	0.005	0.186 (0.053–0.647)
	Low	36.5	43.5	56.5		
p53 status	High	39.7	36	64	0.015	4.781 (1.278–17.884)
	Low	60.3	10.5	89.5		
Ki67 status	High	45.3	25	75	0.71	1.286 (0.390–4.238)
	Low	54.7	20.6	79.4		
Basal phenotype status	Positive	52.7	14.9	85.1	0.26	0.542 (0.185-1.587)
	Negative	47.3	24.4	75.6		

Abbreviation: not applicable. The columns for % pCR and % non-pCR show the response in each category of immunohistochemical variable (positive/negative; high/low). The Pearson's *χ*^2^-significance is presented in the *P*-value column. The Odds ratios with 95% confidence intervals are shown in brackets.
