# Supplementary material for: Topo2α protein expression predicts response to anthracycline combination neo-adjuvant chemotherapy in locally advanced primary breast cancer
Source: Br J Cancer. 2010 Nov 9;103(12):1794–800. doi: 10.1038/sj.bjc.6605960 (PMC3008601; doi:10.1038/sj.bjc.6605960)
Supplement: Supplementary Figure 1 and Table 2 [file 6605960x1.doc]

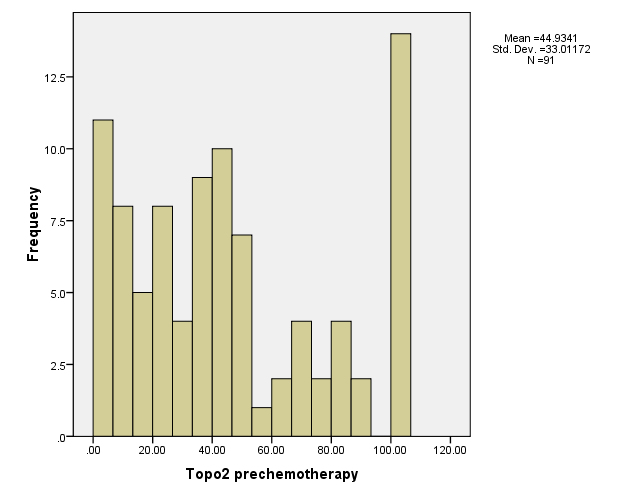


Supplementary Fig1: Histogram showing the distribution of Topo2α scores for the LAPC cohort.

| Parameter | **Variables** | **%** | **%pCR** | **%non-pCR** | **p values** | **Odds Ratio** |
| --- | --- | --- | --- | --- | --- | --- |
| **ER status** | positive | 51.6% | 16.7% | 60.3% | 0.001 | 0.132  (0.35-0.496) |
|  | negative | 48.4% | 83.3% | 39.7% |  |  |
| **PR status** | positive | 36.3% | 25% | 46.8% | 0.116 | 2.636  (0.765-9.080) |
|  | negative | 49.4% | 75% | 53.2% |  |  |
|  | missing | 14.3% | n/a | n/a |  |  |
| **HER2 Status** | positive | 29.7% | 23.5% | 35.4% | 0.354 | 0.562  (0.164-1.923) |
|  | negative | 60.4% | 76.5% | 64.6% |  |  |
|  | missing | 9.9% | n/a | n/a |  |  |
| **Topo2α status** | high | 51.6% | 72.2% | 43.8% | 0.031 | 3.331  (1.076-10.315) |
|  | low | 48.4% | 27.8% | 56.2% |  |  |
| **EGFR status** | high | 63.5% | 33.3% | 72.9% | 0.005 | 0.186  (0.053-0.647) |
|  | low | 36.5% | 66.7% | 27.1% |  |  |
| **p53 status** | high | 39.7% | 69.2% | 32% | 0.015 | 4.781  (1.278-17.884) |
|  | low | 60.3% | 30.8% | 68% |  |  |
| **Ki67 status** | high | 45.2% | 50% | 43.8% | 0.71 | 1.286  (0.390-4.238) |
|  | low | 54.7% | 50% | 56.2% |  |  |
| **Basal phenotype status** | positive | 52.7% | 41.2% | 56.3% | 0.26 | 0.542  (0.185-1.587) |
|  | negative | 47.3% | 58.8% | 43.7% |  |  |

Supplementary Table 2: Immunohistochemistry analysis and correlations with pCR for patients treated with anthracyclines. The columns for % pCR and % non-pCR represent the distribution of the variable within the response category (complete/non-complete). Odds ratios with 95% confidence intervals shown in brackets.
